# Impact of Different Selection Approaches for Identifying Lynch Syndrome-Related Colorectal Cancer Patients: Unity Is Strength

**DOI:** 10.3389/fonc.2022.827822

**Published:** 2022-02-09

**Authors:** Daniele Fanale, Lidia Rita Corsini, Chiara Brando, Alessandra Dimino, Clarissa Filorizzo, Luigi Magrin, Roberta Sciacchitano, Alessia Fiorino, Tancredi Didier Bazan Russo, Valentina Calò, Juan Lucio Iovanna, Edoardo Francini, Antonio Russo, Viviana Bazan

**Affiliations:** ^1^ Department of Surgical, Oncological and Oral Sciences, Section of Medical Oncology, University of Palermo, Palermo, Italy; ^2^ Centre de Recherche en Cancérologie de Marseille (CRCM), INSERM U1068, CNRS UMR 7258, Aix-Marseille Université and Institut Paoli-Calmettes, Parc Scientifique et Technologique de Luminy, Marseille, France; ^3^ Department of Experimental and Clinical Medicine, University of Florence, Florence, Italy; ^4^ Department of Biomedicine, Neuroscience and Advanced Diagnostics (Bi.N.D.), Section of Medical Oncology, University of Palermo, Palermo, Italy

**Keywords:** colorectal cancer, germline mutations, Lynch syndrome, microsatellite instability, mismatch repair genes, *MLH1*, MMR-deficiency, *MSH2*

## Abstract

Lynch syndrome (LS) is an inherited genetic condition associated with increased predisposition to colorectal cancer (CRC) and other tumors and is caused by germline mutations in *Mismatch Repair* (*MMR*) or *EPCAM* genes. The identification of LS carriers is currently based on germline testing of subjects with MMR-deficient (dMMR) tumors or fulfilling clinical criteria, but the most efficient strategies to select patients who should be offered genetic testing are yet not well defined. In order to assess the most suitable selection mode to identify LS-related CRC patients, we retrospectively collected and analyzed all clinical and molecular information of 854 CRC patients, recruited from 2013 to 2021 at the University Hospital Policlinico “P. Giaccone” of Palermo (Italy), 100 of which were selected based on revised Bethesda guidelines, Amsterdam criteria II, or tissue MMR deficiency, and genetically tested for germline variants in LS-susceptibility genes. Our study showed that 32 out of 100 CRC patients harbored germline likely pathogenic/pathogenic variants in *MMR* genes. The analysis of tissue microsatellite instability (MSI) status according to the revised Bethesda guidelines has been to be the best selection approach. However, using different selection approaches as complementary strategies is useful to identify LS carriers, reducing underdiagnosis of this syndrome.

## Introduction

Hereditary Non-Polyposis Colorectal Cancer (HNPCC), more commonly known as Lynch syndrome (LS), is the most prevalent inherited cause of genetic predisposition to colorectal cancer (CRC), by accounting for approximately 1-3% of all newly diagnosed CRC cases (1–3). LS follows an autosomal dominant inheritance pattern with incomplete penetrance (4) and includes, beyond CRC and endometrial cancer (EC), a broad spectrum of LS-associated cancers, with different genetic etiology, risk and tumor characteristics (5–7). Individuals affected by LS have been shown to exhibit an increased lifetime cumulative risk of CRC by up to 80% (25-80%) (7–10).

The LS is caused by germline likely pathogenic/pathogenic variants (LPVs/PVs) in one of the MMR genes, such as *mutL homolog 1* (*MLH1*), *mutS homolog 2* (*MSH2*), *mutS homolog 6* (*MSH6*), and *postmeiotic segregation increased 2* (*PMS2*) (11), or *epithelial cell adhesion molecule* (*EPCAM*) gene, whose deletions at the 3’-end determine *MSH2* epigenetic silencing (12). Generally, about 80-90% of LS has been estimated to be associated with germline *MLH1* and *MSH2* alterations, while 10-20% of cases is attributable to *MSH6* and *PMS2* mutations, and only 3% to *EPCAM* deletions (13). The CRC risk in individuals affected by LS is variable depending on the MMR gene in which the mutation is located, with an earlier age of onset for mutation carriers in *MLH1* or *MSH2* genes compared to carriers in *MSH6* or *PMS2* (7). Generally, LS-associated CRC patients have been observed to have a better clinical outcome than those affected by sporadic CRC (14).

The loss of MMR function determines an increase in DNA replication errors, accumulation of alterations in specific repetitive sequences known as microsatellites, and/or loss of tissue MMR protein expression, resulting in the microsatellite instability (MSI) (15, 16). MSI testing, performed by polymerase chain reaction (PCR) analysis, and/or immunohistochemical (IHC) staining are routinely used in clinical practice for testing MMR deficiency (MMR-D) in CRC (17, 18). Therefore, MSI-high (MSI-H) and MMR-D represent the major molecular hallmarks of LS-associated tumors (19). Since MMR IHC detection is highly correlated with MSI status, both technical approaches, sometimes in combination with somatic *MLH1* promoter hypermethylation and/or somatic *BRAF* V600E mutation analysis, can be used as a reflex testing strategy for identifying LS patients through a subsequent germline MMR testing (20, 21). LS-associated CRCs have usually been shown to be *BRAF wild-type*. However, only 15% of sporadic CRCs is characterized by MSI-H, likely due to epigenetic events which inactivate the MMR system in tumor tissue (22).

Individuals at high-risk of LS can be identified through well-defined clinical criteria, known as Amsterdam criteria II (23) and revised Bethesda guidelines (24) (**Supplementary Table 1**), which take into account the age of tumor onset and family history of cancer. Patients who meet Amsterdam criteria II can directly perform germline genetic testing, regardless of the MMR/MSI status. However, an universal screening able to test the MMR/MSI status in all new diagnosed CRC cases with a greater sensitivity and specificity compared to clinical criteria was recently proposed in order to increase the detection of LS carriers (7). Since a broad phenotypic variability, mainly due to large tumor spectrum and age of onset, has been usually observed in individuals affected by LS and their family members harboring the same germline LPV/PV in MMR genes (25), the choice of the most suitable criteria and optimal screening strategy for selecting subjects to undergo to germline genetic testing are still today debated. For this purpose, we retrospectively harvested and analyzed all clinical and pathological information of CRC patients subjected to germline MMR testing, enrolled at the University Hospital Policlinico “P. Giaccone” of Palermo (Southern Italy), in order to assess the prevalence and typology of different inherited MMR variants detected in LS patients. The aim of our work was mainly to evaluate the most suitable selection mode for identifying LS patients through different approaches, in order to increase diagnostic power of this hereditary disorder.

## Patients and Methods

### Study Population

A retrospective cohort analysis was performed at the “Sicilian Regional Center for the Prevention, Diagnosis and Treatment of Rare and Heredo-Familial Tumors” of the Section of Medical Oncology of University Hospital Policlinico “P. Giaccone” of Palermo. All clinical and pathological information of 854 patients diagnosed with CRC, recruited from May 2013 to June 2021, were retrospectively collected and analyzed, by identifying 100 subjects who underwent genetic counseling and subsequent germline testing for *MMR* (*MLH1*, *MSH2*, *MSH6*, *PMS2*) and *EPCAM* genes for suspected LS, based on the tissue MMR deficiency (detected through IHC), Amsterdam criteria II (23), and revised Bethesda guidelines (24). Therefore, these patients were divided into three subgroups. Patients with familial adenomatous polyposis (FAP) were excluded from our investigation.

All CRC patients undergoing germline genetic testing for LS who showed tumor tissue MMR deficiency (detected through IHC) but did not meet the clinical criteria were included in a separate single group. Patients included through Amsterdam criteria II and revised Bethesda guidelines have been selected regardless of the MMR status assessment, since this data was unknown. Data regarding the MSI and/or MMR status were obtained from the histological reports. Patients with IHC MLH1 deficiency, before being included in a germline testing for *MLH1*, were first tested for somatic *BRAF V600E* mutation or by somatic *MLH1* promoter hypermethylation analysis. Both molecular methods may be used to exclude epigenetically driven inactivation of the *MLH1* gene among patients with MLH1-deficient tumors (26). Only *BRAF*-*wild-type* individuals or without *MLH1* promoter hypermethylation underwent germline *MLH1* genetic testing (4, 20).

Individuals who showed in family a known LS-associated LPV/PV had the opportunity to perform a targeted genetic test. Furthermore, individuals selected through Amsterdam criteria II showed a risk probability ≥5% for LS based on online risk prediction models (MMRpro, PREMM5 and MMRpredict) (4). Genetic counseling was performed by a multidisciplinary group mainly consisting of a geneticist, an oncologist, and a psychologist. The information concerning the personal and familial history of cancer, age at diagnosis, disease stages (I–IV), tumor type and localization, risk factors, MMR/MSI status, and genetic testing results were anonymously recorded for all patients who previously provided a written informed consent.

When a LPV/PV was identified in a patient, the genetic test result was considered informative, whereas it was defined not informative, when no PV or LPV was detected, but their presence could not be excluded, or a variant of uncertain significance (VUS) to which it was not possible to attribute a risk value was detected (27).

Patients harboring a germline LPV/PV in any of analyzed genes were addressed to enhanced screening/surveillance programs and/or risk-reducing surgery strategies by an oncologist with expertise in cancer genetics. Targeted genetic testing was proposed and extended to the first-degree family members of patients harboring a mutation, after providing informed consent (28).

### Sample Collection and Germline Genetic Testing for Lynch Syndrome

Peripheral blood was collected from CRC patients. Genomic DNA was extracted from the peripheral blood using the DNeasy^®^ Blood Kit (QIAGEN, Hilden, Germany) and quantified by Qubit^®^3.0 fluorometer (Thermofisher Scientific, Waltham, MA, USA). Its quality was evaluated by 2100 Bioanalyzer (Agilent Technologies, Santa Clara, CA). Sequencing analysis was performed using Ion 520 Chip (Thermofisher Scientific, Waltham, MA, USA) and Ion Torrent S5 (Thermofisher Scientific, Waltham, MA, USA) instrument. The obtained data was processed with two different software packages called Amplicon Suite (SmartSeq s.r.l.) and Ion Reporter Software v.5.14 (Thermofisher Scientific, Waltham, MA, USA).

The genetic analysis was performed by Next Generation Sequencing (NGS)-based multi-gene panel including predisposition genes involved in LS risk, such as *MLH1*, *MSH2*, *MSH6*, *PMS2*, EPCAM, as previously described (29–31).

The presence of Large Genomic Rearrangements (LGR) in *MMR* and *EPCAM* genes was further tested by Multiplex ligation-dependent probe amplification (MLPA) analysis, using the following SALSA MLPA probemix according to the manufacturer’s instructions (MRC–Holland, Amsterdam, the Netherlands): P003-B2 for *MLH1*, *MSH2* and *EPCAM*; P008-C1 for *PMS2*; and P072-D1 for *MSH6*. Any copy number change in exons 12-15 of *PMS2* was assessed by long-range PCR and subsequent sequencing. Probe amplification products were investigated by capillary electrophoresis using ABI 3130 Genetic Analyzer (Applied Biosystems, Carlsbad, California). Results were evaluated by GeneMapperTM Software Version 3.5 (Applied Biosystems, Carlsbad, California) to determine peak heights and areas and fragment sizes in base pairs (bp), as described previously (32). Positive results were validated with a second analysis using the same kit on another blood sample.

### Sanger Sequencing

LPVs/LPs identified with NGS were confirmed by Sanger sequencing using SeqStudio (Thermofisher Scientific, Waltham, MA, USA) and BigDye Therminator 3.1 Cycle Sequencing Kit (Life Technologies, Carlsbad, CA, USA), according to the manufacturers’ protocols (28).

### Genetic Variant Classification

The detected genetic variants were classified according to criteria established by Evidence-based Network for the Interpretation of Germline Mutant Alleles (ENIGMA) consortium (https://enigmaconsortium.org/), and IARC recommendations (33), and divided into five classes: benign (class I), likely benign (class II), VUS (class III), likely pathogenic (class IV), and pathogenic (class V). Several databases were used for the identification and classification of genetic variants, such as ClinVar, LOVD and Varsome.

The detected variants were named based on the recommendations for the description of sequence variants provided by the Human Genome Variation Society (HGVS), whose nomenclature was approved by the Human Variome Project (HVP) and Human Genome Organization (HUGO) (34).

## Results

### Clinico-pathological Features of CRC Patients Undergoing Genetic Testing for Lynch Syndrome

**Figure 1:**
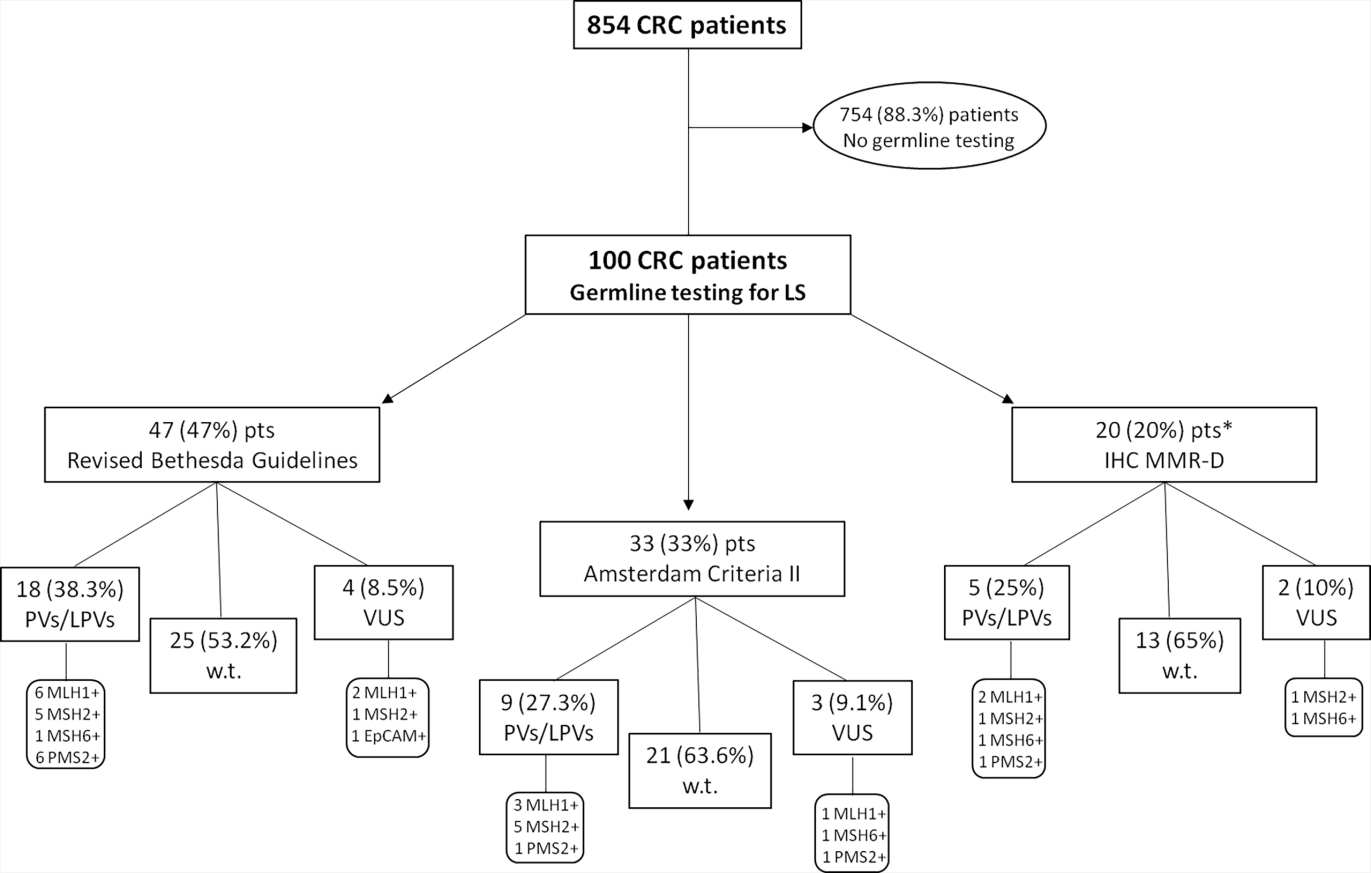
Flow chart reporting the study design. One hundred CRC patients underwent genetic counseling and germline testing for *MMR* and *EPCAM* genes for suspected Lynch Syndrome, and divided into three subgroups on the basis of the following criteria: tumor MMR deficiency (detected through IHC), Amsterdam criteria II, and revised Bethesda guidelines. *This subgroup includes only IHC *MLH1*-deficient patients negatively tested for somatic *BRAF V600E* mutation (*BRAF*-*wild-type*) and/or without *MLH1* promoter hypermethylation. CRC, Colorectal Cancer; IHC, immunohistochemistry; MMR-D, Mismatch Repair Deficiency; LPV, Likely Pathogenic Variant; Pts, Patients; PV, Pathogenic Variant; VUS, Variant of Uncertain Significance; w.t., wild-type.

A retrospective analysis of the clinico-pathological and molecular information from 854 CRC patients, enrolled from May 2013 to June 2021, was performed at the “Sicilian Regional Center for the Prevention, Diagnosis and Treatment of Rare and Heredo-Familial Tumors” of the Section of Medical Oncology of University Hospital Policlinico “P. Giaccone” of Palermo. One hundred out of 854 investigated patients underwent genetic counseling and subsequent germline testing for *MMR* and *EPCAM* genes for suspected LS, and divided into three subgroups on the basis of the following criteria: tumor MMR deficiency (detected through IHC), Amsterdam criteria II, and revised Bethesda guidelines (**Figure 1**).

The clinico-pathological features of 100 studied CRC patients (65 of which females and 35 males) are summarized in **Table 1**. The average age of the CRC diagnosis was 53 years. Ninety-two patients showed single CRC and, among them, 40% had a tumor localized in sigma-rectum, 34% in left colon and 18% in right colon. Among LS-related neoplasms, endometrial and breast cancers were the most frequently observed tumors (12% and 8%, respectively). As regards the family history, 97% of patients had a family history of cancer, predominantly CRC (50%).

**Table 1:** Clinico-pathological characteristics of CRC patients underwent germline genetic testing for Lynch Syndrome.

CRC (n = 100)	NUMBER (%)
**AGE AT DIAGNOSIS (years)**	
<50 years	39 (39)
≥50 years	61 (61)
**AVERAGE AGE (range)**	52.92 (25-85)
**SEX**	
M	35 (35)
F	65 (65)
**TUMOR TYPE AND LOCATION**	
**Single CRC**	92 (92)
LEFT COLON	34 (34)
RIGHT COLON	18 (18)
SIGMA-RECTUM	40 (40)
**Multiple CRC**	8 (8)
**OTHER ASSOCIATED TUMORS**	
Endometrial cancer	12 (12)
Ovarian cancer	2 (2)
Breast cancer	8 (8)
Urothelial cancer	2 (2)
Renal cancer	2 (2)
Parotid cancer	1 (1)
Glioblastoma	1 (1)
Thyroid cancer	1 (1)
Nasopharynx cancer	1 (1)
Prostate cancer	1 (1)
Gastric cancer	1 (1)
**NO ASSOCIATED TUMORS**	68 (68)
**FAMILY HISTORY OF CANCER**	97 (97)
CRC	50 (50)
non-CRC	11 (11)
CRC and non-CRC cancer	36 (36)
**NO FAMILY HISTORY**	3 (3)
**GENETIC TESTING RESULTS FOR LS**	
LPV/PV	32 (32)
* MLH1*	11 (34.4)
* MSH2*	11 (34.4)
* MSH6*	2 (6.2)
* PMS2*	8 (25)
* EPCAM*	0 (0)
VUS	9 (9)
* MLH1*	3 (33.4)
* MSH2*	2 (22.2)
* MSH6*	2 (22.2)
* PMS2*	1 (11.1)
* EPCAM*	1 (11.1)
* Wild-type*	59 (59)

CRC, Colorectal Cancer; LPV, Likely Pathogenic Variant; LS, Lynch Syndrome; PV, Pathogenic variant; VUS, Variant of uncertain significance.

### Impact of Different Patient Selection Methods for Increasing Lynch Syndrome Diagnosis

In order to investigate the impact and usefulness of different selection approaches and their discriminating power in the identification of LS-related CRC patients, we collected genetic testing data of all individuals appropriately selected for germline screening through different methods, because the use of only clinical and computational criteria often results in the loss of a substantial percentage of affected individuals. Almost half of the CRC patients (47%) undergoing germline testing for LS was recruited based on the revised Bethesda guidelines, 33% according to Amsterdam criteria II, and 20% based on IHC MMR deficiency (**Figure 1**). No Amsterdam criteria II-selected subjects was overlapping with revised Bethesda guidelines-selected patients, because most of these (32 out of 47; 68.1%) harbored only one CRC diagnosed before the age of 50 years, while 8 (17%) individuals exhibited multiple (synchronous or metachronous) CRCs or LS-associated tumors regardless of age, and, finally, 7 (14.9%) CRC patients had only one first-degree relative with LS-related cancer diagnosed before age 50 years.

All 100 CRC probands, who met the previously established criteria, after appropriate genetic counseling, were genetically tested for germline variants in different LS-associated susceptibility genes, such as *MLH1*, *MSH2*, *MSH6, PMS2* and *EPCAM*. The mutational screening of the investigated study cohort showed that 59 out of 100 probands carried germline *MMR* benign/likely benign variants (*MMR*-*w*.*t*.), whereas 32 patients harbored a germline *MMR* LPV/PV (*MMR*-positive), and 9 subjects were carriers of germline *MMR* VUS (class III). In particular, germline *MMR* LPVs/PVs were detected in 18 (38.3%) out of 47 CRC patients selected by means of revised Bethesda guidelines, 9 (27.3%) out of 33 subjects screened for Amsterdam criteria II, and 5 (25%) out of 20 patients recruited for negative IHC MMR testing. Overall, considering 32 germline *MMR*-positive CRC probands, the highest number of LS diagnoses comes from the cluster of CRC patients selected by revised Bethesda guidelines (18/32; 56.3%), secondly from the subgroup of individuals fulfilling Amsterdam criteria II (9/32; 28.1%), and, to a lesser extent, from subset of tissue MMR-deficient (dMMR) subjects (5/32; 15.6%). This information could be useful and interesting to establish what is the best selective approach for genetically testing CRC patients with suspected LS.

**Figure 2:**
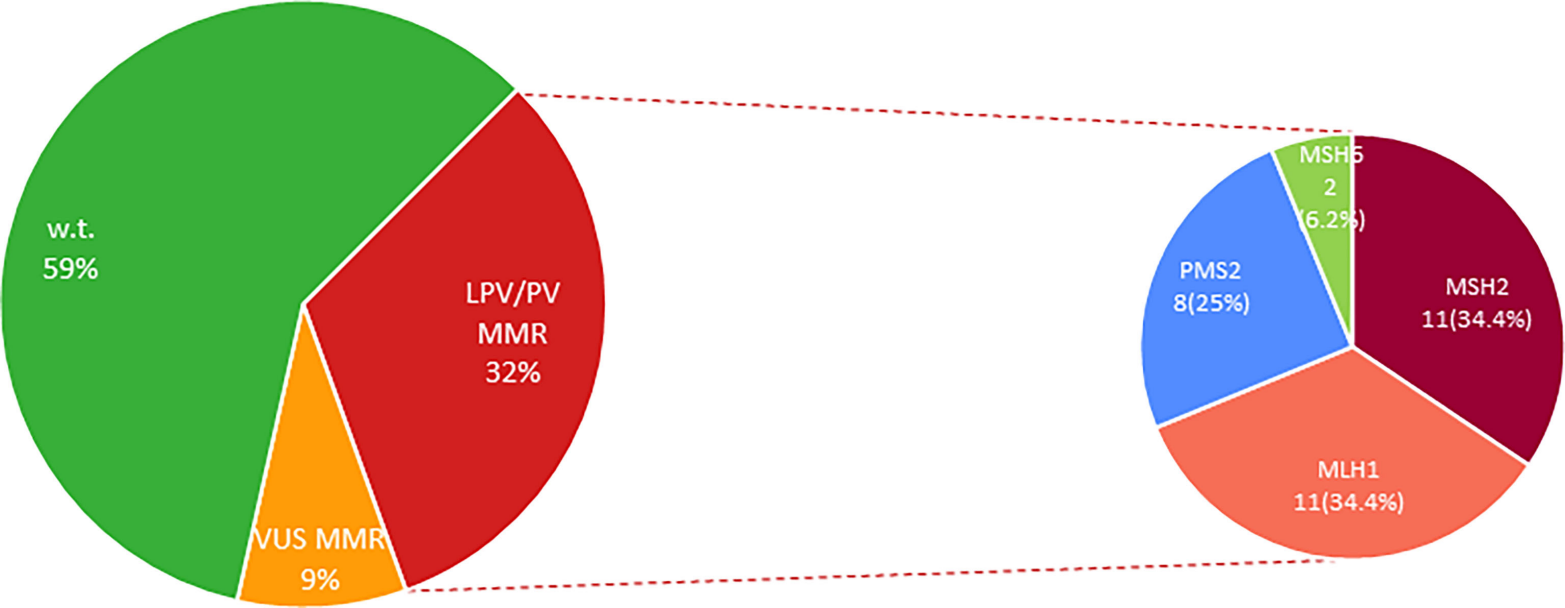
Percentage distribution of MMR genes altered in LS-associated CRC patients. MMR, Mismatch Repair genes; LPV, Likely Pathogenic Variant; Pts, Patients; PV, Pathogenic Variant; VUS, Variant of Uncertain Significance; w.t., wild-type.

Our analysis also revealed that 11 (34.4%) out of 32 CRC patients positively tested for *MMR* genes have been shown to harbor germline *MLH1* PVs, other 11 (34.4%) subjects carried germline *MSH2* LPVs/PVs, 8 (25%) were carriers of germline *PMS2* PVs, and 2 (6.2%) individuals showed germline *MSH6* PVs (**Figure 2** and **Table 2**). No germline pathogenic alteration was detected in *EPCAM* gene. The *MMR* VUS detected in 9 CRC patients were distributed as follows: three in *MLH1*, two in *MSH2* and *PMS2*, respectively, and only one in *MSH6* and *EPCAM*, respectively (**Figure 2** and **Table 3**).

**Table 2:** Germline *MMR* likely pathogenic/pathogenic variants detected in LS-related CRC patients.

Gene	Nucleotide change HGVS nomenclature	Amino acid change	Variant type	ClinVar classification	VarSome	No. patients
*MLH1*	c.454-_?545+?del	–	FS	PV	–	1
	c.208-3C>G	–	IVS	LPV	LPV	2
	c.1852_1854delAAG	p.Lys618del	FS	PV	PV	1
	c.(1558 + 1_1559-1)_(*193_)?del(p)? (del ex14-19)	–	LGR	–	–	1
	c.1975C>T	p.Arg659Ter	NS	PV	PV	2
	c.1195dup	p.Arg399fs	FS	–	PV	1
	c.199G>C	p.Gly67Arg	M	PV	PV	3
*MSH2*	c.212-1G>A	–	IVS	PV	PV	2
	c.2459dup	p.Gly820LysfsTer117	FS	–	PV	1
	c.976_977del	p.Leu326GlyfsTer6	FS	–	PV	1
	c.(1076 + 1_1077-1)_(1276 + 1_1277-1)del (del ex7)	p.Leu360Lysfs*16	LGR	PV	–	1
	c.942+344_1076+7988del9655 (del ex6)	–	LGR	–	–	1
	c.484G>A	p.Gly162Arg	M	PV	PV	2
	c.2740del	p.Glu914LysfsTer2	FS	LPV	PV	3
*MSH6*	c.3261dup	p.Phe1088LeufsTer5	FS	PV	PV	1
	c.3G>C	p.Met1Ile	M	PV	PV	1
*PMS2*	c.137G>T	p.Ser46Ile	M	LPV	PV	3
	c.1A>T	p.Met1Leu	M	PV	PV	1
	c.2182_2184delinsG	p.Thr728AlafsTer7	FS	CIP	PV	1
	c.2T>C	p.Met1Thr	M	LPV/LP	PV	1
	c.1927C>T	p.Gln643Ter	NS	PV	PV	2

IVS, intronic variants; FS, frameshift variant; M, missense variant; NS, nonsense variant; LGR, large genomic rearrangement; PV, pathogenic variant; LPV, likely pathogenic variant; CIP, conflicting interpretations of pathogenicity.

**Table 3:** Germline *MMR* variants of uncertain significance detected in LS-related CRC patients.

Gene	Nucleotide change HGVS nomenclature	Amino acid change	Variant type	ClinVar classification	VarSome	PolyPhen-2/SIFT	No. patients
*MLH1*	c.1154G>A	p.Arg385His	M	CIP	LPV	Light/Damaging	1
	c.1277A>T	p.Gln426Leu	M	VUS	VUS/LPV	Tolerated/Light	2
*MSH2*	c.435T>G	p.Ile145Met	M	CIP	VUS/LPV	Light/Tolerated, Damaging	2
*MSH6*	c.1385C>T	p.Pro462Leu	M	VUS	VUS/LPV	Light/Damaging	1
	c.3674C>T	p.Thr1225Met	M	VUS	VUS/LPV	Light/Damaging	1
*PMS2*	c.2249G>A	p.Gly750Asp	M	CIP	VUS/LPV	Light/Damaging	1
*EPCAM*	c.583C>G	p.Leu195Val	M	VUS	VUS	Light/Tolerated	1

VUS, variants of uncertain significance; CIP, conflicting interpretations of pathogenicity; PV, pathogenic variant; LPV, likely pathogenic variant; M, missense.

Alterations in *MLH1*, *MSH2* or *PMS2* genes, respectively, were equally distributed among the revised Bethesda guidelines-selected *MMR*-positive patients, whereas more than half of Amsterdam criteria II-selected *MMR*-positive patients showed LPVs/PVs in *MSH2* gene. In addition, globally considering the 32 *MMR*-mutated patients, revised Bethesda guidelines-selected subjects showed the highest mutation rate in *MLH1* and *PMS2* genes (12 out of 32 patients; 37.5%), while the same mutation frequency (15.6%) for *MSH2* gene was observed in individuals enrolled based on revised Bethesda guidelines and Amsterdam criteria II, respectively.

In our population cohort, *MSH2* alterations have been observed to be mainly harbored by CRC women who had developed also endometrial cancer. Interestingly, most of *PMS2* PV carriers showed, beyond CRC, some cases of associated breast cancer, supporting the recent hypothesis that this neoplasm could also be included in the LS tumor spectrum, as already highlighted by other studies (35–39). This could explain the higher number of *PMS2* PVs (8/32; 25%) detected in our patient cohort compared to data reported in literature.

In general, the mutational analysis did not show a more prevalent LPV/PV than others detected in our study population, probably due to the low number of total identified mutations. However, interestingly the *MSH2* LPV named c.2740del (p.Glu914fs) has previously been described in other Sicilian families from South-eastern coast of Sicily, as already reported by Cavallaro et al. (40). Finally, the *PMS2* variant c.137G>T (p.Ser46Ile), detected in three CRC probands of our study cohort, has been reported in literature as a Caucasian founder mutation (41, 42).

## Discussion

LS is an inherited genetic condition associated with an increased broad spectrum cancer risk, mainly conferring a genetic predisposition to CRC and EC. The main clinico-pathological features of LS are personal and family history of LS-related cancers, autosomal dominant inheritance, earlier age of CRC onset (~ 45 years) compared to sporadic CRC cases (~ 69 years), greater localization of the tumor in the right colon, presence of multiple CRCs, and poorly differentiated tumors (43, 44).

The identification of LS carriers is currently based on the germline *MMR* and *EPCAM* testing of individuals with dMMR tumors or fulfilling clinical criteria, but the most efficient and sensitive strategies to select patients among CRC probands to whom it should be offered are yet not well defined (2). In most cases, LS remains underdiagnosed, since it has been estimated that 98% of carriers of gene alterations predictive of LS have yet to be identified, causing the lack of implementation of efficient preventive strategies able to reduce the tumor incidence. In fact, intensive CRC surveillance by colonoscopy and prophylactic gynecological surgery have been shown to decrease mortality rate of LS patients (2). Furthermore, improving the diagnosis rate has become essential, because LS patients can now benefit from new treatments such as immunotherapy (45).

Several studies highlighted that Amsterdam criteria II and revised Bethesda guidelines exhibit some limitations causing the loss of a clinically significant proportion of LS carriers (46, 47). In particular, Amsterdam criteria II involve the clinical evaluation of the patient and his family for CRC and other LS-related tumors with very high specificity (98%), but low sensitivity (22–42%), because more than 50% of LS families are not included in these criteria (44, 48). Bethesda revised guidelines, instead, allow to identify individuals at risk for LS without a strong family history who deserve a genetic analysis through tumor MSI and/or IHC testing, in order to further select those patients who should be genetically tested for germline mutations (49). These guidelines are more sensitive (82-95%) but less specific (77-93%) compared to the Amsterdam criteria (4). A high correlation between the MSI and IHC data was observed, but IHC analysis often is the preferred option for a wide MSI screening, because protein staining is technically easier to carry out compared to DNA analysis (49, 50). Both techniques show comparable sensitivity and specificity. However, while the sensitivity of IHC analysis is 83%, independently of the involved *MMR* gene, instead that of MSI testing is dependent on the *MMR* gene in which the mutation is located (80-91% for *MLH1* or *MSH2* alterations, and 55-77% for *MSH6* or *PMS2* mutations). The IHC and MSI testing exhibit almost the same specificity (89% *vs* 90%, respectively) (4, 51).

Since several evidence showed that Amsterdam criteria II are not reliable in terms of sensitivity/specificity, probably due to poor accuracy and consistency of the collected information about the family history, some authors have proposed to remove this component from the preliminary selection approaches of individuals newly diagnosed with CRC (48).

Universal MMR screening among CRC probands has been shown to have a greater sensitivity and accuracy in the identification of individuals with LS and more clinically actionable germline mutations compared to other multiple selection approaches, although the increase in the diagnostic power is modest due to a lower specificity (2, 52). However, a large-scale immunohistochemical characterization of CRCs for the assessment of MMR expression is needed in order to increase the specificity of this selection approach (53).

Since the identification rate of LS carriers needs to be improved, today, the debate about the choice of the best approach to identify LS high-risk patients who should be offered germline testing still remains open. In fact, until now, no well-defined guidelines have been written to provide the most appropriate approach for selecting the most suitable patients for germline testing. For this purpose, in our investigation, different selection approaches based on germline MMR testing performed on patients harboring dMMR tumors detected by IHC, or fulfilling the Amsterdam criteria II or at least 1 criterion of the revised Bethesda guidelines were compared with each other, in order to assess the best screening strategies useful to minimize the number of CRC patients with undiagnosed LS. None of the patients belonging to the three studied groups was overlapping with each other. Increasing the diagnostic power of LS through a suitable screening procedure is useful also for the unaffected family members of identified LS patients. Indeed, the higher the number of LS diagnosis, the higher the number of at risk family members who may be genetically tested and, eventually, undertake intensive surveillance pathways and cancer risk-reducing personalized preventive strategies, in order to decrease morbidity and mortality related to LS. Furthermore, patients harboring MSI-H cancers showed a better clinical outcome compared to those with microsatellite stability. Therefore, assessing the MMR status by MSI or IHC analysis of all CRC subjects has prognostic implications and may be useful to decide the most suitable therapy (54, 55). However, larger study cohorts are needed in order to improve the diagnosis rate of LS.

In our work, we retrospectively collected and analyzed all clinical and molecular information of 100 CRC patients who have been genetically tested for germline variants in different LS-related susceptibility genes. This study was also aimed to assess whether it was useful to offer a MMR analysis by IHC to all CRC patients, regardless their cancer family history and age at diagnosis.

Our investigation showed that almost half of the CRC patients undergoing germline testing was enrolled based on the revised Bethesda guidelines, whereas a lower percentage of probands was genetically tested because of a MMR deficiency detected by IHC. Probably, this lower number may be due to the fact that, to date, not all CRCs undergo broad molecular screening through MMR IHC. Overall, 32 patients have been shown to harbor a germline *MMR* LPV/PV, more than half of which (56.3%) were selected by revised Bethesda guidelines, 28.1% by individuals fulfilling Amsterdam criteria II, and 15.6% by subjects with dMMR tumors. The relatively high percentage of *MMR-w.t.* patients identified only based on personal and familial history of LS-associated tumors (Amsterdam criteria II), in addition to the lower sensitivity of the selection strategy, may probably be due to the presence of uninvestigated germline mutations in other CRC susceptibility genes such as *MUTYH*, *POLE*, *POLD1*, *PTEN*, *STK11*, *TP53*, *SMAD4*, *BMPR1A* (56–58).

Therefore, in light of these results, revised Bethesda guidelines seem to have a higher discriminating power in the identification of LS-related CRC patients compared to other selective approaches. Interestingly, by only selecting patients based on clinical criteria, in the absence of data collected from tumor MMR screening by IHC, a certain proportion (5/854; 0.6%) of LS carriers would have been lost. However, this data is underestimated because not all investigated CRC patients underwent broad molecular screening through MMR IHC. This hypothesis is logically supported by the significant percentage (5/20; 25%) of LS carriers identified in the subset of tissue dMMR subjects. Combining the mutational data both from revised Bethesda guidelines and IHC-based MMR deficiency screening, it is observed that it would be more useful to assess tumor MSI/dMMR status in all CRC patients as a optimal selection approach, in support of the recent findings showing the validity of universal screening as a true driver in the identification and diagnosis of subjects with LS (59, 60). Furthermore, since these molecular tests show different sensitivity and specificity, both approaches should be viewed as complementary strategies.

In conclusion, our work showed that a larger selection of CRCs through multiple approaches may help us to stratify a higher portion of LS-associated CRC patients who may benefit from the screening programs, active surveillance strategies, or cancer risk-reducing surgery interventions, where necessary. Also, this data could provide helpful suggestions and insights that could contribute to the improvement of the current guidelines, greatly reducing the underdiagnosis of this inherited genetic condition. Consequently, this information could have a strong clinical impact on the choice of the best therapeutic option by clinicians, by allowing the selection of subgroups of CRC patients affected by LS who may benefit from immunotherapy treatments (61).

However, it should be noted that our study in addressing the proposed aims shows some limitations, such as small sample size and missing data on MMR status, suggesting that greater information about MMR status of CRCs and a larger study cohort to determine more accurate detection rates for LS are needed.

## Data Availability Statement

The datasets presented in this article are not readily available because of ethical and privacy reasons. Requests to access the datasets should be directed to antonio.russo@usa.net and viviana.bazan@unipa.it.

## Ethics Statement

The studies involving human participants were reviewed and approved by Ethics Committee of the University Hospital A.O.U.P. “P. Giaccone” of Palermo. The patients/participants provided their written informed consent to participate in this study.

## Author Contributions

Conceptualization: DF, LC, and CB. Data curation: DF, LC, CB, AD, CF, LM, RS, AF, and EF. Formal analysis: DF, LC, AD, CF, TB, and VC. Investigation: LM, RS, and JI. Methodology: DF and CB. Project administration: AR and VB. Supervision: JI, EF, AR, and VB. Critical revision of the manuscript: EF; Writing—original draft: DF, LC, and CB. All authors contributed to the article and approved the submitted version.

## Conflict of Interest

The authors declare that the research was conducted in the absence of any commercial or financial relationships that could be construed as a potential conflict of interest.

## Publisher’s Note

All claims expressed in this article are solely those of the authors and do not necessarily represent those of their affiliated organizations, or those of the publisher, the editors and the reviewers. Any product that may be evaluated in this article, or claim that may be made by its manufacturer, is not guaranteed or endorsed by the publisher.
