# Supplementary material for: Impact of Different Selection Approaches for Identifying Lynch Syndrome-Related Colorectal Cancer Patients: Unity Is Strength
Source: Front Oncol. 2022 Feb 9;12:827822. doi: 10.3389/fonc.2022.827822 (PMC8864140; doi:10.3389/fonc.2022.827822)
Supplement: Supplementary file 1 [file Table_1.docx]

**Supplementary Table 1.** Amsterdam criteria II and revised Bethesda guidelines used as strategies for identifying individuals with suspected Lynch Syndrome to undergo to MMR genetic testing.

| Amsterdam II criteria |
| --- |
| Three or more relatives with a histologically verified Lynch sindrome-related tumors (colorectal, endometrial, small bowel, ureter or renal pelvis) of which: |
| - a relative should be a first-degree relative of the other two; |
| - cancer involving at 2 least generations; |
| - one or more cancers must be diagnosed before the age of 50 years; |
| - familial adenomatous polyposis should be excluded. |
| Revised Bethesda Guidelines |
| Colorectal cancer diagnosed in a patient younger than 50 years; |
| Presence of synchronous or metachronous colorectal cancer or other Lynch syndrome-related tumors, regardless of age; |
| Colorectal cancer with microsatellite instability-high histology; |
| Colorectal cancer diagnosed in a patient with one or more first-degree relatives with lynch syndrome-related cancer, with one of the tumors diagnosed before the age of 50; |
| Colorectal cancer diagnosed in a patient with two or more first- or second-degree relatives with Lynch syndrome-related cancers regardless of age. |
